# Elongation of the Cervix Uteri

**Published:** 1909-05-15

**Authors:** 


### Gynæcology.

### ELONGATION OF THE CERVIX UTERI.

Elongation of the supra-vaginal portion of the cervix uteri is a common condition, and plays an important part in cases of prolapse of the vaginal walls and incidentally in descent of the uterus itself. Elongation of the vaginal portion of the cervix is uncommon and is a congenital malformation in its strict sense, for those cases in which there is hypertrophy of the vaginal portion associated with bilateral lacerations and chronic endocervicitis are not strictly elongations. These latter conditions are really a hyperplasia of all the tissues of the injured cervix, and the elongation present is apparent, not real. True elongation of the portio vaginalis exists from birth, and may be discovered for the first time after marriage or in a virgin if some other symptom calls for examination. Of two such cases seen recently by the writer, one occurred in a married woman who had had one child, and the other occurred in a virgin who acquired just sufficient descent of the uterus to make the elongated cervix appear at the vaginal orifice. In both these cases the portio vaginalis was elongated to the extent of one and a half inches of projection from the vaginal roof. In all respects, except length, there was nothing abnormal; there was no evidence of an inflammatory lesion, and the tissues appeared quite healthy on section. In both cases the reason for seeking advice was the appearance of the os uteri at the vaginal entrance on account of a slight degree of descent of the uterus, acquired in the one case as a result of labour and subinvolution, in the other (the virgin) as a result of heavy work and overstrain. There can be no mistaking these conditions for any other form of elongation, for on pushing up the whole uterus into its proper place in the pelvis, the portio vaginalis projects from the vaginal fornices in a manner which at once makes the diagnosis clear.

When the elongation occurs in the supra-vaginal portion of the cervix the condition is always acquired; by far the commonest cause, if not the only one, is the drag of the vaginal walls upon their vaginal attachments in cases of cystocele, with sometimes rectocele in addition. The important question arises, why is it that in some cases cystocele and rectocele produce prolapse of the uterus without any cervical elongation, in others produce some prolapse with some cervical elongation, and yet in others produce elongation without appreciable prolapse of the uterus? The answer in any particular case is not always easy to formulate, but it must primarily depend on the degree of looseness of the uterine attachments. It is easy to see that if the uterus is only loosely held in the pelvis, any drag on the cervix by the vaginal walls will produce descent of the whole organ without any tendency to elongate the cervix. We see this more often in old women, in whom the vaginal walls are likely to become completely everted, carrying the uterus with them. On the other hand, in younger or middle-aged women, it is possible for the bladder to form a cystocele, and yet the uterus may be held just sufficiently firmly in its place to prevent easy descent. In such cases the drag of a constant cystocele may slowly cause extension of the supra-vaginal cervix, whilst the fundus uteri only very gradually descends. These are the cases of moderate uterine descent with so much elongation of the supra-vaginal cervix as to make the internal measurement of the uterus reach four or five inches, all due to supra-vaginal elongation of the cervix. These are the common cases met with clinically; the third class of case in which cystocele causes supra-vaginal elongation without any uterine descent is very rare. One such case known to the writer occurred in a young woman who had had ventro-fixation done for uterine prolapse. This held the fundus closely to the abdominal wall, but did not prevent the cystocele present from constantly exerting traction on the cervix and slowly elongating it until the os uteri appeared at the vulva. Such a case was an object-lesson not only in the ætiology of supra-vaginal elongation, but also in the futility of ventro-fixation *alone* as a cure for prolapse of the uterus.

Pathologically, elongation is usually accompanied by some hypertrophy of the cervix, for although the diameter of the elongated portion is smaller than normal, it is not so small as it should be if the elongation were mere stretching. There must be some compensatory tissue growth, and as a rule this is a fibrous hyperplasia. It is difficult to say what the effect upon the bladder is in these cases, for very few frozen sections of such conditions are in existence. But when we think how loose the attachment of the bladder usually is to the uterus and how much the bladder projects downwards in a bad cystocele, it is probable that the bladder descends as a whole with the uterus, and that its actual attachment to the uterus is not stretched. We do not find an undue amount of bladder attached to an elongated cervix if we wish to strip off the bladder. The symptoms of an elongated cervix cannot be differentiated from those caused by prolapse of the uterus; it is only on examination that we can say whether there is more prolapse than cervical elongation or vice versa. It is unnecessary to use the uterine sound for this purpose, for the great length of the uterus and its comparatively small diameter and bulk can be easily appreciated on making a bi-manual examination after pushing the cervix back and replacing the vaginal vault as far as possible with the fingers. The sound should only be used to define accurately the length of the uterus just before operating on such a case when the vagina and external parts have been made aseptic. When the uterus has been pushed up into its normal place in the pelvis and the vaginal fornices do not descend, it is easy to distinguish between supra-vaginal and true elongation of the vaginal portion, for under these conditions the latter may still be seen to project definitely below the fornices.

The treatment of any form of cervical elongation must be amputation in the first instance, combined with measures to prevent a recurrence of the causes of that elongation.

